# Green Chiral HPLC Method for Lumefantrine Analysis: Method Development, Greenness Assessment, and Computational Studies

**DOI:** 10.1002/chir.70122

**Published:** 2026-07-16

**Authors:** Amanda Mohr, Gustavo Machado das Neves, Érika Segala, Vera Lucia Eifler‐Lima, Martin Steppe

**Affiliations:** ^1^ Laboratório de Controle de Qualidade Farmacêutico (LCQFar), Faculdade de Farmácia Universidade Federal do Rio Grande do Sul Porto Alegre Rio Grande do Sul Brazil; ^2^ Laboratório de Síntese Orgânica Medicinal (LaSOM), Faculdade de Farmácia Universidade Federal do Rio Grande do Sul Porto Alegre Rio Grande do Sul Brazil; ^3^ Faculdade de Farmácia Universidade Federal do Rio Grande do Sul Porto Alegre Rio Grande do Sul Brazil

**Keywords:** absolute configuration, chiral, enantioseparation, ethanol, green methods, lumefantrine, molecular docking

## Abstract

Currently, greening analytical methods have become of great interest to ensure operators' and environment health. Chiral analysis can commonly employ hazardous chemicals and large quantities of waste. In this context, this study presents a green chiral high‐performance liquid chromatography (HPLC) method for the determination of lumefantrine (LUME) enantiomers. Chromatographic enantioseparation was achieved on a Chiralpak ad column with a mobile phase composed of ethanol at a flow rate of 1 mL/min. The effects of column temperature on the retention of the enantiomers were investigated, and the analysis temperature was set at 35°C. The method greenness profile was assessed using the Analytical GREEnness (AGREE) and Analytical Green Star Area (AGSA) metrics by which the method proved to be eco‐friendly. The method was validated, showing adequate selectivity, linearity, precision, accuracy, and robustness over the range of 80–120 μg/mL per enantiomer. Electronic circular dichroism (ECD) analysis proposed that the first eluted peak was the R‐enantiomer and the second eluted peak was the S‐enantiomer. Molecular docking studies suggested that LUME may interact mainly through van der Waals and π‐interactions on the Chiralpak ad. A green method was successfully developed for the chiral separation of LUME. The proposed work contributes to more environmentally friendly chiral approaches in pharmaceutical analysis.

## Graphical abstract

A green chiral HPLC method was developed and validated for the determination of lumefantrine enantiomers. The method employs ethanol as a mobile phase as a sustainable and efficient alternative to hexane. The chiral recognition mechanism and elution order of the enantiomers were investigated using electronic circular dichroism and molecular docking studies.

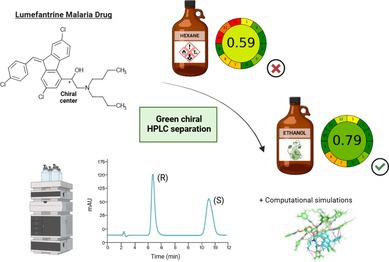

## 1 Introduction

In recent decades, the use of hazardous chemicals in analytical processes has raised significant environmental concerns. This issue is particularly relevant in the pharmaceutical industry, where numerous analyses are performed daily. More than 50% of drugs are chiral and require enantioselective methodologies for their separation, pharmacokinetics and toxicological studies, and quality control. Among the techniques employed for this purpose, high‐performance liquid chromatography (HPLC) with a chiral stationary phase (CSP) stands out as the most commonly used [1, 2, 3].

Based on the principles of green analytical chemistry (GAC), several strategies can be implemented to achieve greener HPLC enantioselective methods. One strategy is to reduce solvent consumption by decreasing the particle size or dimensions of the CSP. However, this approach often requires more advanced and expensive instruments. Another effective strategy is to replace traditionally used hazardous solvents, such as hexane, dichloromethane, and acetonitrile (ACN), with environmentally greener alternatives [4, 5, 6].

Several alternative solvents, including ethyl lactate, glycerol, cyrene, acetone, propylene glycol, and dimethyl carbonate, have been evaluated as greener options for liquid chromatography [7, 8, 9, 10]. Despite their potential, technical challenges such as low water solubility, high viscosity, elevated system pressure, and high UV cutoff still have limited their adoption in chiral separations. Given these limitations, bio‐based ethanol (EtOH) remains one of the most viable greener alternatives for chiral separation. For instance, Varfaj et al. [11] successfully separated some chiral sulfoxides using polysaccharide‐based CSPs with a mobile phase of EtOH/2‐propanol (80:20). Similarly, Mhammad et al. [12] demonstrated the efficiency of EtOH with the additive diethylamine for the enantioseparation of crizotinib, further confirming the robustness of ethanol‐based systems for sustainable chiral resolutions.

In order to evaluate and compare the environmental impact of analytical methodologies, several green metrics tools have been developed over the years. These include the National Environmental Method Index (NEMI), Analytical Eco‐scale, Modified Green Analytical Procedure Index (MoGAPI), and Analytical GREEnness (AGREE) [13, 14, 15]. Among these, AGREE is one of the most widely used metrics, providing a recognized color‐coded pictogram and a final score based on the 12 principles of GAC [16, 17]. More recently, the Analytical Green Star Area (AGSA) was introduced as a new metric aligned with the 12 principles. AGSA stands out by incorporating method classification and enhanced resistance to user bias, aiming to provide a more standardized and objective evaluation of analytical procedures [18].

**FIGURE 1:**
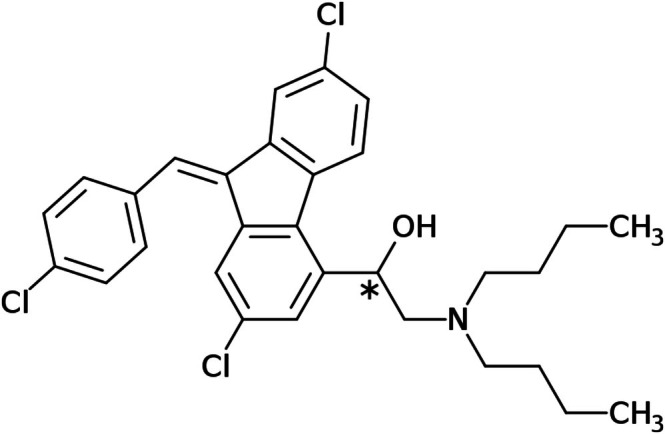
Chemical structure of lumefantrine. *Chiral center.

Lumefantrine (LUME) is a chiral drug (Figure 1) used in combination with artemether for the treatment of acute uncomplicated malaria infections caused by *Plasmodium falciparum* [19]. Studies suggested the occurrence of pharmacokinetic stereoselectivity of LUME, with plasma concentration data favoring higher levels of the (+)‐LUME enantiomer compared to its antipode, regardless of the racemate's dosing route [20, 21]. This pharmacological behavior encourages further study into a potential chiral switch, a usual strategy to the development of a single enantiomer drug from the previously approved racemate, which could lead to simpler and more selective pharmacological profiles. From this perspective, analytical methods capable of separating the LUME enantiomers are crucial for supporting enantioselectivity studies and therapeutic optimization [22, 23, 24].

Despite the critical need for enantioselective methods, a literature survey reveals only one reported HPLC method for the separation and quantification of LUME enantiomers in dosage form. The method employs a polysaccharide‐based CSP under the normal phase mode, with a mobile phase containing 97% hexane, a well‐known hazardous chemical [25]. Chiral separations performed under normal phase commonly employ polar hazardous solvents, leading to important issues in terms of ecological impact and operator safety. Therefore, incorporating green HPLC methods becomes highly desirable to replace or reduce the use of hazardous solvents in analytical procedures [26, 27, 28].

In this context, the aim of this work was to develop a green enantioselective HPLC method for the quantification of LUME enantiomers. The overall greenness profile of the proposed method was assessed against the reported one using the AGREE and AGSA metrics. Due to the lack of availability of pure enantiomers of LUME, electronic circular dichroism (ECD) analysis was conducted to propose the absolute configuration and elution order of the enantiomers. Additionally, molecular docking studies were performed to suggest the interactions between the drug and the CSPs tested.

## 2 Experimental

### 2.1 Materials

LUME reference material with an assigned purity of 98% was supplied by Sigma Aldrich, USA. Artemether 20‐mg and lumefantrine 120‐mg tablets (Macleods) were provided by the Ministry of Health of Brazil. The CSPs assessed were Chirobiotic T (150 × 4.6 mm, 5 μm) based on glycopeptide teicoplanin (Astec, USA), Chirobiotic V (150 × 4.6 mm, 5 μm) based on glycopeptide vancomycin (Astec, USA), and Chiralpak ad (150 × 4.6 mm, 5 μm) based on amylose tris(3,5‐dimethylphenylcarbamate) (Daicel Chiral Technologies, China).

Methanol (MeOH), ACN, and 1‐propanol (PrOH) were obtained from Merck, Germany. EtOH was obtained from Panreac, Spain. Ammonium acetate and glacial acetic acid were acquired from Sigma Aldrich, USA. Ammonium formate was obtained from Neon, Brazil. The tablets' excipients were obtained by Synth, Brazil (colloidal silicon dioxide, croscarmellose sodium, hypromellose, magnesium stearate, microcrystalline cellulose, and polysorbate 80). Ultrapure water was produced using a Direct‐Q apparatus, Millipore, France.

### 2.2 Equipment

The HPLC analyses were conducted on an HPLC Agilent 1200 series (Agilent, Germany), equipped with a G1311A quaternary pump, ALS‐G1329A autosampler, TCC‐G1316A column oven, G1315B photodiode array detector (PDA), and ChemStation software for instrument control, data acquisition, and data analysis.

The ECD analyses were conducted on a Jasco J‐815 spectrometer (Jasco, Japan), with a 10‐mm pathlength cell at a 25°C temperature analysis. The spectra were registered from 200 to 800 nm with intervals of 1 nm. The method was set to a spectral bandwidth of 1 nm, a scanning speed of 100 nm/min, a time constant of 2 s, and 3 accumulations. The samples were diluted in EtOH and the spectra were background corrected with EtOH. The UV spectrum of LUME, obtained by the HPLC‐PDA system in the same solvent, is provided in Figure S1.

### 2.3 Sample Preparation

LUME tablets stock solution of 500 μg/mL (the equivalent of 250 μg/mL per enantiomer) was prepared by weighing the equivalent of 25 mg of LUME finely crushed tablets and diluting it in a volumetric flask containing 50 mL of EtOH. LUME reference material stock solution of 500 μg/mL (the equivalent of 250 μg/mL per enantiomer) was prepared by weighing 50 mg of LUME reference material and diluting it in a volumetric flask containing 100 mL of EtOH. Both stock solutions were sonicated in an ultrasonic bath for 20 min to ensure complete solubilization. Working solutions were prepared daily by diluting appropriate aliquots of the stock solutions in EtOH. All solutions were filtered through a 0.45‐μm membrane filter and stored at a temperature of 2°C–8°C, protected from light. Under these conditions, the solutions remained stable for at least 30 days with no relevant changes in the chromatographic response.

### 2.4 Chromatographic Conditions

The final optimized method conditions were CSP Chiralpak ad, a mobile phase of EtOH, flow rate of 1.0 mL/min, column oven temperature of 35°C, injection volume of 10 μL, and detection wavelength set at 264 nm.

### 2.5 Green Method Assessment

The overall greenness profile of the proposed method was assessed against the reported method using the AGREE and AGSA metrics. The pictograms were generated using the open‐source softwares available at https://mostwiedzy.pl/AGREE (version 0.5, 2020) and https://fotouhmansour.github.io/AGSA/ [17, 18].

### 2.6 Method Validation

The method was validated according to the International Council for Harmonisation of Technical Requirements for Pharmaceuticals for Human Use (ICH) Q2(R2) guideline on validation of analytical procedures, to assess its selectivity, range, precision, accuracy, and robustness [29].

Selectivity was demonstrated through comparative analyses of LUME reference material (100 μg/mL per enantiomer), LUME tablet samples (100 μg/mL per enantiomer), and a placebo solution composed of all the LUME tablets' excipients. The absence of interference from the tablets' excipients in the enantiomers quantification was confirmed using the ChemStation software peak purity tool.

The range was evaluated using a linear calibration model, where the enantiomer peaks' areas (response) were plotted as a function of their corresponding concentrations. The standard curve was prepared at five concentrations in the range of 80–120 μg/mL per enantiomer of LUME reference material. Each concentration level was prepared independently in triplicate (*n* = 15). Analysis of variance (ANOVA) was conducted using Excel to assess the correlation coefficient, the *y*‐intercept and slope of the regression line, and the significance of the linear regression and lack of fit. The absence of outliers of the residuals was graphically interpreted, and the homoscedasticity of the data was assessed by the Cochran C‐test. Statistical significance was set at 5%.

The detection limit (DL) and quantitation limit (QL) were estimated based on the standard deviation of the *y*‐intercept and slope of the standard curve, using factors of 3.3 and 10, respectively.

Precision was assessed by repeatability and intermediate precision. Repeatability was determined by analyzing six independent LUME samples (100 μg/mL per enantiomer) on the same day. Intermediate precision was determined by analyzing six independent LUME samples (100 μg/mL per enantiomer) on two consecutive days and by two analysts. The results were expressed by relative standard deviation (RSD%), with an acceptance criterion of RSD% < 2%.

Accuracy was evaluated through a recovery study. A placebo solution was spiked with LUME reference material at three concentration levels per enantiomer (90, 100, and 110 μg/mL), each level in triplicate. The results were expressed as the mean percent recovery of the known added amount of the reference material in the placebo. The acceptance criterion was a recovery range of 95%–105%.

Robustness was verified by performing deliberate variations in the chromatographic conditions and comparing them with the nominal conditions. The variations included flow rate (±0.02 mL/min), detection wavelength (±1 nm), and column oven temperature (±0.2°C). LUME enantiomer contents were statistically analyzed using Student's *t*‐test. Statistical significance was set at 5%.

### 2.7 Absolute (R) and (S) Configuration and Enantiomeric Elution Order

The absolute configuration and elution order of the enantiomers were proposed by simulating the theoretical ECD spectrum of the (R)‐LUME enantiomer and comparing it with the experimental ECD spectra measures of the eluted peaks. A semi‐preparative enantioseparation was performed using the developed method in order to isolate the LUME enantiomers and submit them to the experimental ECD analysis.

The theoretical ECD spectrum was simulated according Pescitelli and Bruhn [30] using the tridimensional structure of (R)‐LUME obtained as follows: (1) The SMILES notation was obtained from Pubchem [31] and the geometry was optimized through molecular mechanics followed by semiempirical function (MMFF + PM6) approach using Wavefunction Spartan'14 v.1.1.8 [32], and this refined structure was also used in molecular docking studies; (2) the optimized structure was utilized as starting point for conformational search employing MMFF at Wavefunction Spartan'14 with a maximum search of 10,000 conformers; (3) the resultant conformers had their geometry further optimized in ORCA 6.1.0 [33, 34] through the usage of density functional theory (DFT) method with the composite functional PBEh‐3c; (4) the conformers with relative energy lower than 3 kcal/mol were selected for the next steps; (5) the selected conformers were further optimized employing DFT using the Becke, 3‐parameters, Lee‐Yang‐Parr, functional with D4 dispersion correction (B3LYP‐D4) with def2‐TZVP as basis set, and the conductor‐like polarizable continuum model (CPCM) of EtOH was chosen as implicit solvation model and TightSCF as convergence criteria; (6) the Boltzmann distribution was applied at 298 K to select 1% of the conformer population to frequency and spectra calculation; (7) the frequency calculation of the best conformers was achieved using DFT with B3LYP‐D4/def2‐TZVP, CPCM model of EtOH, and TightSCF as convergence criteria; (8) the spectra calculation was achieved using the simplified time‐dependent density functional theory (sTD‐DFT) [35] with *ω*B97X‐D3/def2‐TZVP, CPCM model of EtOH and TightSCF convergence criteria. The resulting output files were converted for ORCA 6.0.1 format by an in‐house python script, and the calculated ECD spectra were analyzed and processed in SpecDis tool 1.71 [36], in which the enantiomers spectra were obtained according to the Boltzmann distribution of the most representative conformations and compared to the experimental data. The obtained spectra were not shifted and the sigma was set in 0.30 eV. Further figure enhancements were achieved in Inkscape 1.3.

### 2.8 Molecular Docking

Molecular docking studies were conducted to suggest possible interactions between lumefantrine and the CSPs tested. The molecular docking protocol followed a previously described methodology [37]. The structures of LUME used in docking studies were optimized using the MMFF + PM6 protocol as reported in the previous section. The constituents from Chirobiotic T (PDBID: 3mgb, chain C containing teicoplanin), Chirobiotic V (PDBID: 1aa5, chains A, B, and C corresponding to vancomycin), and Chiralpak ad [38] were preprocessed in UCSF Chimera [39] through the DockPrep module in order to include hydrogens and Gasteiger charges. The molecular docking was processed in vacuum conditions in CCDC GOLD 5.2 [40] using ChemPLP as scoring functions and default parameters. The grid locations and sizes are reported in Table S1. The interaction profile was analyzed in Discovery Studio Visualizer 2025 [41], and the figures were obtained in Pymol 3.0 [42].

## 3 Results and Discussion

### 3.1 Method Development

Initially, the choice of solvent for sample preparation was investigated. LUME is reported to be practically insoluble in water, soluble in dichloromethane, and slightly soluble in MeOH [43]. Previous achiral methods for the quantification of LUME usually employ chloroform and dichloromethane for sample dilution or involve the addition of acids to improve solubilization [44, 45, 46]. Experimental tests revealed that LUME can be effectively dissolved in EtOH at concentrations of up to 500 μg/mL. Therefore, taking into account the toxicity of all solvents, EtOH was selected for sample preparation.

The development of the method was conducted in order to achieve the greenest enantioselective method. Considering the good results achieved by [25] with the amylose‐based CSP Chiralpak ad, this CSP was chosen to be further studied, along with the macrocyclic glycopeptide‐based Chirobiotic T and V CSPs. As a polysaccharide chiral selector, Chiralpak ad is recognized for its efficiency and reproducibility, using its helical (alpha‐1,4) architecture for chiral recognition. In contrast, macrocyclic glycopeptides CSPs offer the unique advantage of possessing ionizable groups. This functionality enables ionic interactions, which are absent in polysaccharide selectors, and complements hydrogen bonding and π‐π interactions to provide broader selectivity [47, 48].

The normal separation mode was excluded due to the high toxicity of its solvents, and the reversed‐phase was the first mode selected to be investigated. The injection volume of all experiments was 10 μL, and the detection wavelength was set at 264 nm. Mobile phases in the reversed‐phase mode generally consist of a combination of organic solvents and an aqueous component. The organic solvents EtOH, PrOH, MeOH, and ACN were investigated. Although ACN and MeOH are widely used in reversed‐phase mode, they present some environmental impact and health safety issues, as shown in Table 1. Among alternative solvents, EtOH and PrOH are considered to be green solvents. EtOH, in particular, stands out as one of the best green solvents due to its bio‐based source, appropriate toxicity and ecotoxicity profiles, and safety of handling by operators [52, 53].

**TABLE 1:** Comparison between GlaxoSmithKline (GSK), Pfizer, and CHEM21 solvent selection guides of commonly used HPLC solvents [49, 50, 51].

	GSK	Pfizer	CHEM21
Water			
Ethanol			
1‐propanol			
Methanol			
Acetonitrile			
Acetic acid			
Formic acid			
Triethylamine			
Hexane			
Dichloromethane			

*Note:* Solvent classification is indicated by color: green (recommended), yellow (problematic), red (hazardous), and gray (no data available).

The aqueous component was investigated with the addition of ammonium acetate and ammonium formate buffer at different ionic strengths and pH adjustment with acetic acid in the range of 3–7, considering that the pKa of LUME is 8.73 [54]. Several ratios of organic solvents and aqueous components were tested, but no enantioseparation was obtained in the three CSPs explored. This lack of resolution across the tested pH range might be related to the protonation state of LUME. In acidic aqueous phases, the molecule is likely protonated and more polar, potentially hindering its stereoselective interaction with the selectors. This charged state may reduce affinity for the neutral grooves of the polysaccharide CSP and may prevent a proper fit within the macrocyclic cavities of the glycopeptide selectors. Consequently, it is hypothesized that the protonation of the analyte prevents the specific spatial stabilization required for enantiorecognition in reversed‐phase mode [47, 48].

Because pH variations did not enable enantioresolution, the addition of buffers and pH modifiers was discontinued. Instead, a simpler binary system of organic solvent and water was investigated to evaluate whether the elimination of ionic competition from buffer salts could facilitate the interaction between LUME and the CSPs. Under these conditions, it was observed that increased water content resulted in longer retention times for LUME, leading to very extended runs (> 60 min). With the proportion of water reduced to 10%, it was possible to obtain a partial separation of the enantiomers with the combination of EtOH and the Chiralpak ad column in less than 60 min. From an ecological perspective, a reversed‐phase approach using water and EtOH would be a favorable option. However, for a highly hydrophobic molecule like LUME (logP = 9.19) this strategy proved impractical, as the long retention times would lead to excessive solvent and energy consumption [12, 54].

**FIGURE 2:**
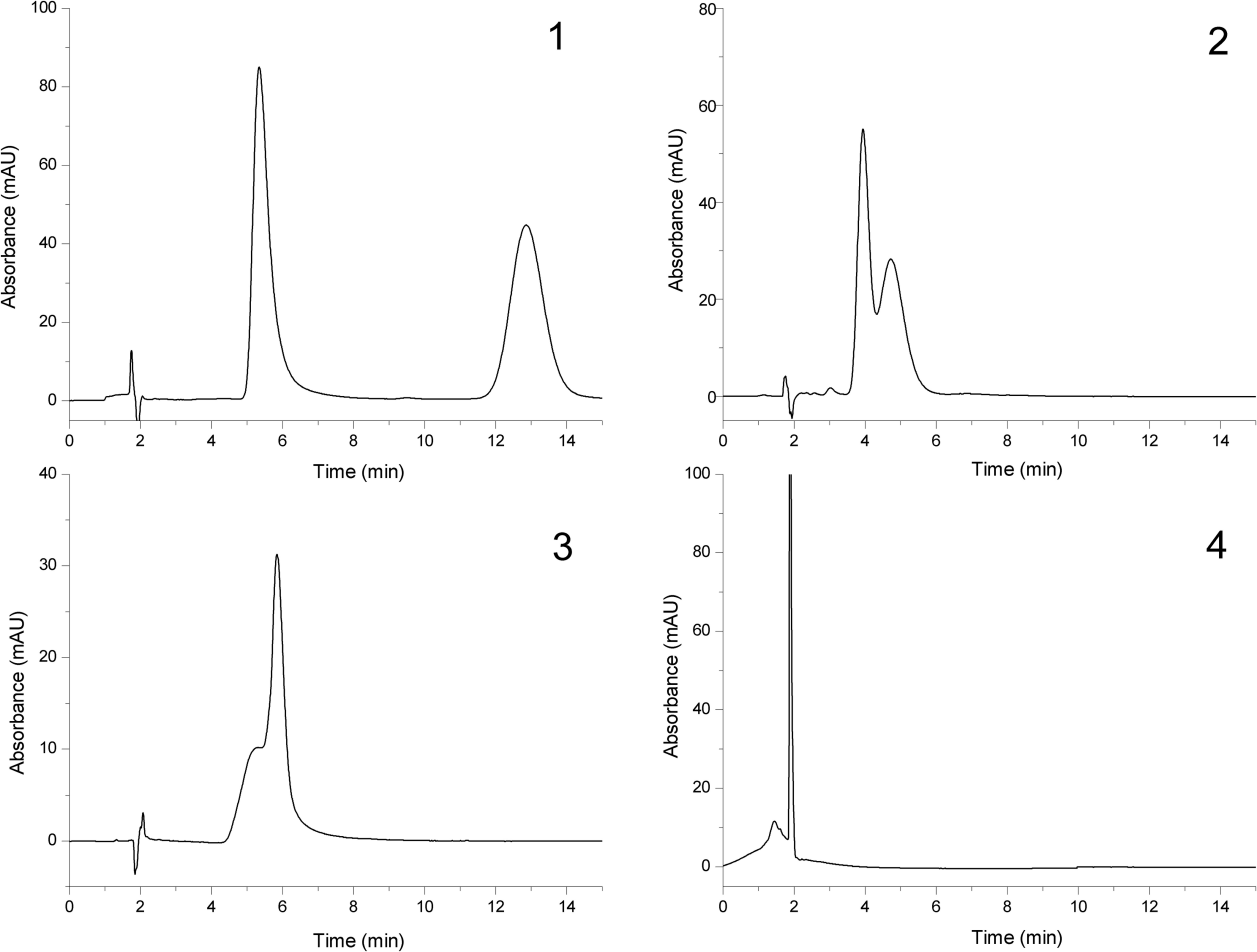
Comparison of LUME enantioresolution in polar organic mode with different solvents: (1) ethanol, (2) methanol, (3) 1‐propanol, and (4) acetonitrile. Chromatographic conditions: Chiralpak ad CSP*,* flow rate of 1.0 mL/min, temperature 25°C, injection volume of 10 μL, detection wavelength of 264 nm.

Therefore, the organic solvents EtOH, PrOH, MeOH, and ACN were tested at 100%, without water content, also known as the polar organic mode. With the Chirobiotic T and V CSPs, no enantioresolution was observed with any of the solvents. With the Chiralpak ad, ACN did not result in enantioseparation. Partial separation was observed with MeOH and PrOH, and adequate resolution (> 1.5) was achieved with EtOH at a flow rate of 1 mL/min (Figure 2). This outcome could potentially be attributed to the more open and helical structure of the polysaccharide‐based CSP, which may have allowed for a better accommodation of the LUME structure compared to the macrocyclic phases [55].

The differences in enantioselectivity observed with the Chiralpak ad column underline the critical role of the mobile phase's chemical properties in the chiral recognition process. The lack of resolution with ACN is likely due to its aprotic nature, which may restrict the hydrogen‐bonding interactions often essential for chiral discrimination in this mode. Regarding the alcohols, the results suggest a sensitive dependence on the balance between polarity and steric factors. The high polarity of MeOH might have caused competition for the interaction sites, whereas the increased molecular volume of 1‐PrOH likely hindered the geometric fit of LUME structure. Thus, EtOH appears to have provided an optimal environment, potentially offering the ideal balance of interaction strength and steric compatibility required for the successful enantioseparation [56].

**FIGURE 3:**
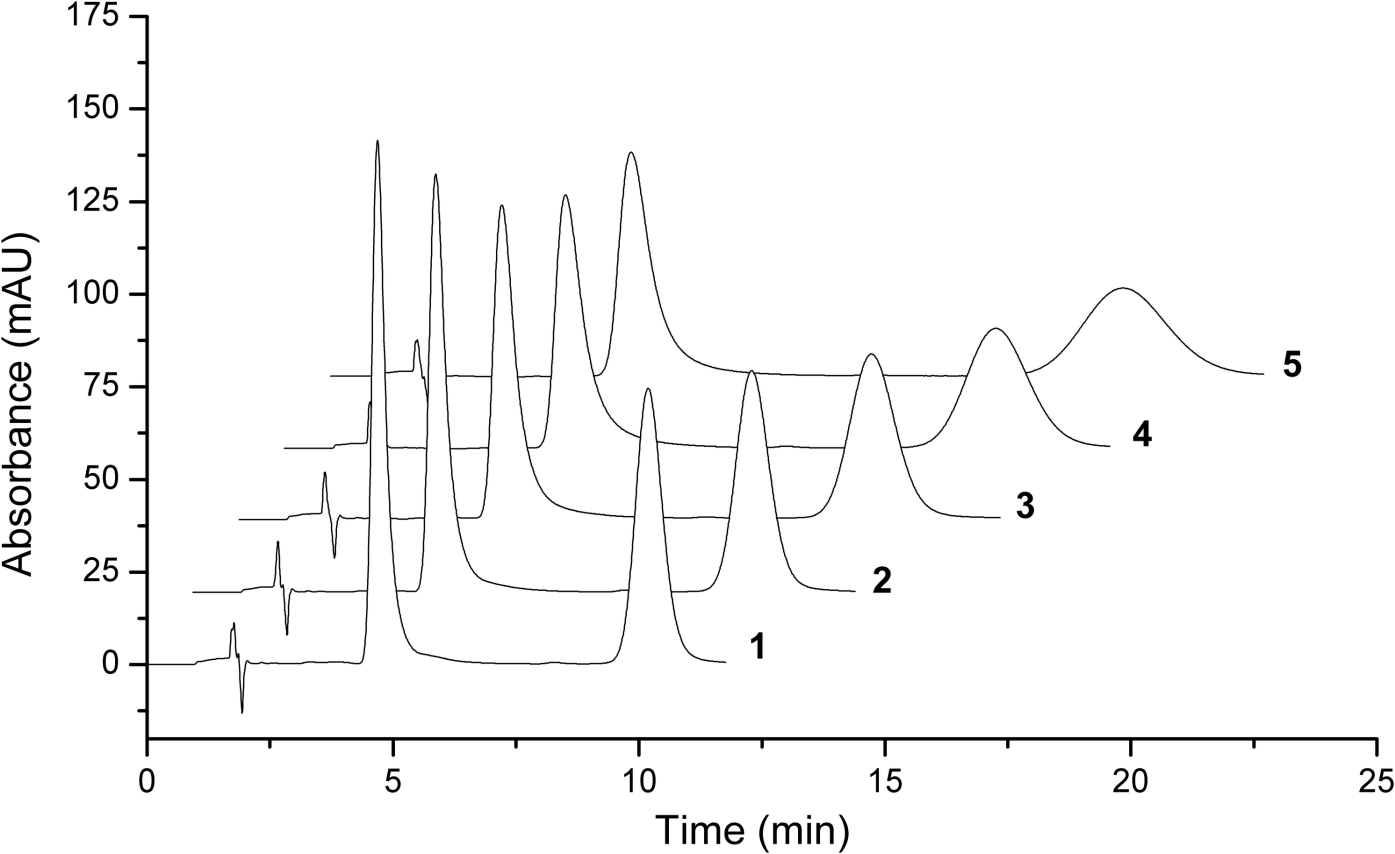
Effect of column temperature on the LUME enantiomers retention. (1) 35°C; (2) 30°C; (3) 25°C; (4) 20°C; and (5) 15°C. Chromatographic conditions: Chiralpak ad CSP, mobile phase EtOH, flow rate of 1.0 mL/min, injection volume of 10 μL, detection wavelength of 264 nm.

**FIGURE 4:**
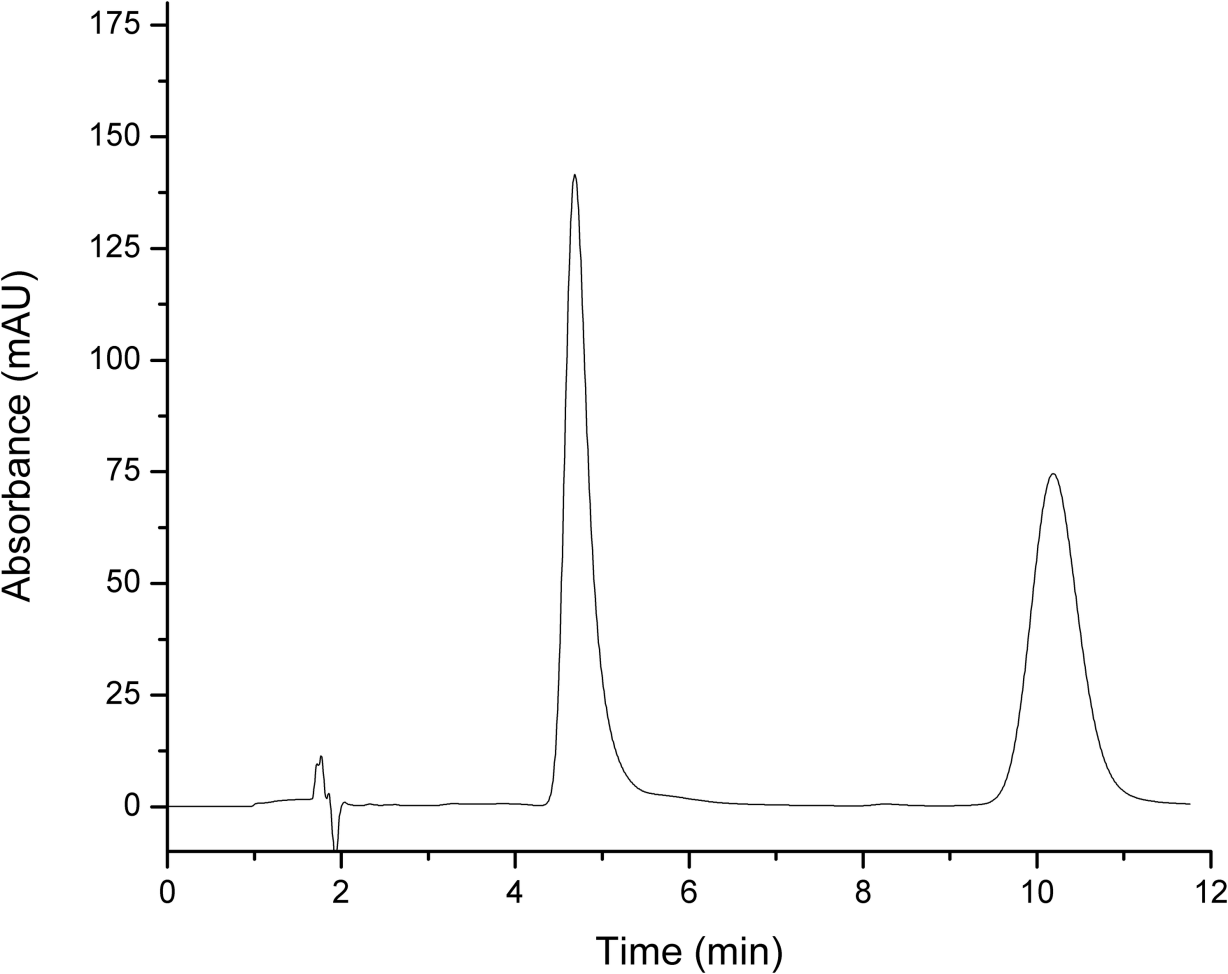
Illustrative chromatogram of the developed method for the enantioseparation of LUME (100 μg/mL per enantiomer). Chromatographic conditions: Chiralpak ad CSP, mobile phase EtOH, flow rate of 1.0 mL/min, temperature 35°C, injection volume of 10 μL, detection wavelength of 264 nm.

To further explore the chiral enantioseparation behavior, the Chiralpak ad CSP temperature was evaluated in the range of 15°C–35°C (Figure 3). The temperature of 35° provided the shortest retention time along with adequate resolution. Finally, the mobile phase composed of 100% EtOH at a flow rate of 1 mL/min in the Chiralpak ad column at 35°C was found to be the greener HPLC condition for LUME enantioseparation, with a resolution of 5.88 (Figure 4). It is important to emphasize that the presence of EtOH alone does not inherently guarantee a green method. The necessity of cosolvents or additives, which are often required even in polar organic or normal phase modes, must be carefully evaluated. Some literature strategies for pharmaceutical enantioseparations, such as those reported for lurasidone, may use ethanol as a green component but still need to rely on high proportions of nonsustainable solvents like hexane (92:8, v/v) [57]. By contrast, our method prioritizes a simplified, monosolvent system to minimize both waste and chemical toxicity.

### 3.2 Green Method Assessment

The AGREE and AGSA metrics were applied to evaluate and compare the greenness profiles of the developed method and the reported method by [25]. Both metrics revealed a similar profile for both methods regarding Principles 1–9. In the AGREE assessment, both methods received green scores for Principles 2, 3, 4, 5, and 9 and yellow‐orange scores for Principles 1, 7, and 8 due to the off‐line nature of the analysis, amount of waste generated, and limited sample throughput, respectively. In contrast, the AGSA provided a more granular evaluation of these shared limitations. For instance, Principles 3, 4, and 5 were identified as limitations in AGSA because, in both methods, measurements are not performed in situ, only partial process integration is used, and procedures are semi‐automated.

A distinction between the metrics was observed in Principle 7. Although AGREE assigned a yellow‐orange score to both methods for waste management, AGSA highlighted a distinction: the proposed method was credited for waste recycling and minimization efforts, whereas the reported method followed only standard disposal procedures. This demonstrates how the AGSA assessment can reveal nuances of greenness that might be averaged out in the AGREE score.

The main difference between the methodologies is evidenced in Principles 10, 11, and 12, where the proposed method achieves green scores and the reported method receives red scores. Regarding Principle 10, in the reported method, none of the chemicals used are from bio‐based sources, whereas in the proposed method, EtOH can be obtained from bio‐based sources. In Principle 11, hexane is categorized as a hazardous and toxic solvent, whereas EtOH in the proposed method is associated with lower toxicity. Finally, Principle 12 concerns operator safety, where the reported method involves high‐risk procedures requiring extensive personal protective equipment, whereas the proposed method involves low‐risk procedures.

**FIGURE 5:**
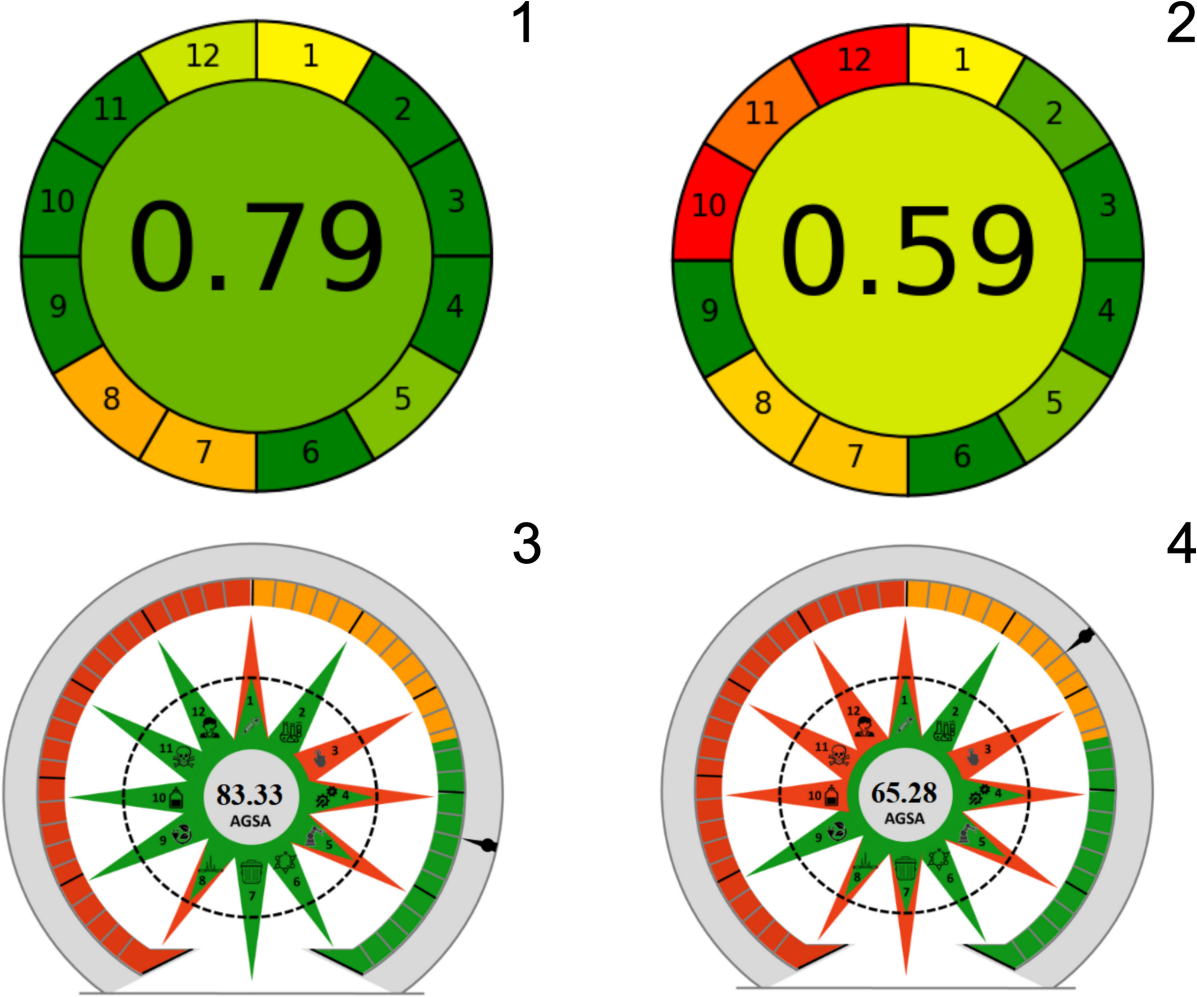
Greenness assessment: (1) AGREE—proposed method, (2) AGREE—reported method, (3) AGSA—proposed method, and (4) AGSA—reported method.

These findings, reflected in the superior final scores of the proposed method (AGREE: 0.79; AGSA: 83.33) compared to the reported method (AGREE: 0.59; AGSA: 65.28), confirm the green and eco‐friendly nature of the developed method (Figure 5). The AGSA emerged as a valuable novel metric, offering a straightforward and user‐friendly assessment that is resistant to bias by eliminating the user‐defined weights used in AGREE. Furthermore, the AGSA provided a clear method classification, categorizing the proposed method as green and the reported method as orange.

It is worth noting that some principle scores remained constrained in both methodologies by the inherent technical requirements of the HPLC platform, such as the instrument's remote location, the current level of automation and miniaturization, and specific energy demands. The replacement of hexane with ethanol significantly reduced the toxicity and improved the overall safety profile of the proposed method. Nevertheless, there is still room for further sustainability enhancements in the proposed method, particularly regarding analysis time, waste generation, and analytical throughput. These aspects could be further optimized by using columns with smaller particle sizes or reduced dimensions along with compatible instrumentation. To the best of our knowledge, this stands as the first method to provide a sustainable alternative for LUME analysis.

### 3.3 Method Validation

A validation study was conducted to demonstrate that the developed analytical method is fit for the intended purpose, evaluating the following performance characteristics: selectivity, range, precision, accuracy, and robustness [29]. For clarity, the first eluted enantiomer peak was labeled as E1 and the second eluted enantiomer peak as E2.

No interference from the tablet excipients was observed in the quantification of LUME enantiomers in the tablets and reference samples, confirming the method's selectivity.

A linear relationship between each enantiomer peak area and its concentration was obtained over the range of 80 to 120 μg/mL. The correlation coefficient, the *y*‐intercepts and slopes of the regression lines are provided in Table 2. For both enantiomers, ANOVA confirmed the linear regression (E1: *F*calc = 2446 > *F*crit_(1, 13)_ = 4.67; E2: *F*calc = 2706 > *F*crit_(1, 13)_ = 4.67) and lack of fit (E1: *F*calc = 0.15 < *F*crit_(3, 10)_ = 3.71; E2: *F*calc = 0.31 < *F*crit_(3, 10)_ = 3.71). No outliers of the residuals were observed, and the data were homoscedastic. The estimated DL and QL of each enantiomer are provided in Table 2.

**TABLE 2:** Validation data of range, precision, and accuracy of the developed method for the determination of LUME enantiomers.

	E1	E2
Range		
Standard curve	80–120 μg/mL	80–120 μg/mL
Slope	30.28	30.77
SE of slope	0.61	0.59
Intercept	56.13	16.05
SE of intercept	61.85	59.73
Correlation coefficient	0.9973	0.9976
DL	6.74 μg/mL	6.41 μg/mL
QL	20.42 μg/mL	19.41 μg/mL
Precision (RSD%)		
Repeatability (*n* = 6)	0.50	0.51
Intermediate precision (*n* = 18)	1.97	1.93
Accuracy (% of recovery)		
Low level	96.00	96.12
Medium level	95.40	96.57
High level	95.09	95.38

*Note:* All analyses were performed in duplicate.

Abbreviations: DL = detection limit, E1 = first‐eluted enantiomer, E2 = second‐eluted enantiomer, QL = quantitation limit, RSD = relative standard deviation, SE = standard error.

The RSD% values of the repeatability and intermediate precision were below 2%, confirming the method precision. The mean percent of recoveries of LUME reference material at the three evaluated concentration levels was in the acceptance range of 95%–105%, confirming the method accuracy (Table 2). The deliberate variations performed in the chromatographic conditions were not statistically significant, demonstrating the robustness of the method. Such robustness is particularly crucial for chiral separations, as it ensures reliable resolution and quantification of enantiomers despite minor changes in operational conditions.

### 3.4 Absolute (R) and (S) Configuration and Enantiomeric Elution Order

**FIGURE 6:**
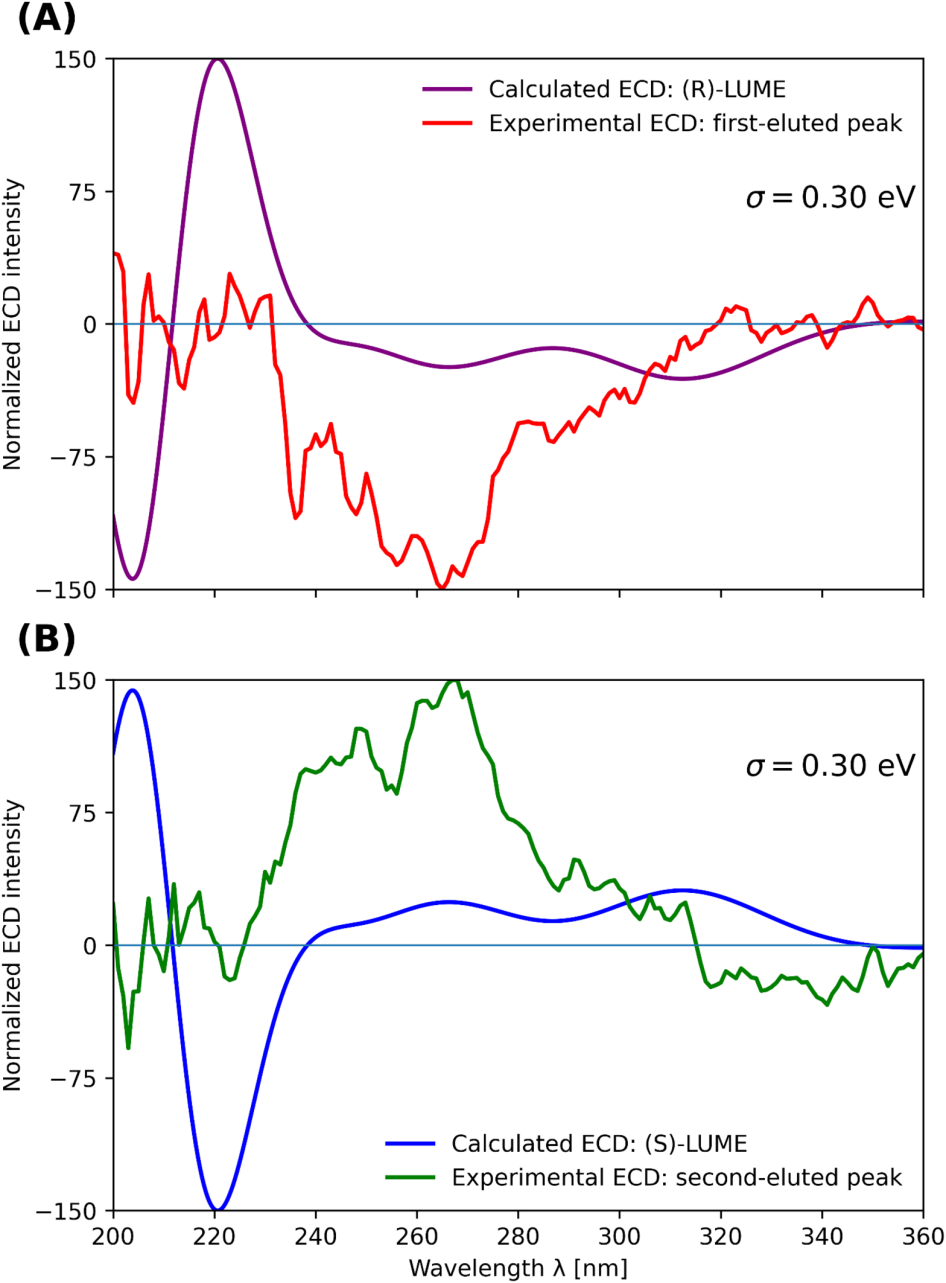
Comparison of theoretical and experimental ECD spectra of LUME enantiomers.

Figure 6 compares the experimental and theoretical ECD spectra for the LUME enantiomers. Despite the noise observed in the experimental data, likely associated with the instrumental acquisition parameters, the spectra exhibited the expected mirror‐image enantiomeric pattern. The comparison between experimental and theoretical ECD data is a well‐established approach for the absolute configuration assignment of chiral compounds [58, 59, 60], providing the basis for the proposed assignment. The theoretical ECD spectrum was generated from 20 conformations of (R)‐LUME with 30 excited states calculated for each conformer. All conformers produced by this workflow showed no imaginary frequencies, indicating that they correspond to true minima rather than saddle points or transition states.

LUME contains 67 atoms (35 heavy atoms—C, N, O, and Cl—and 32 hydrogens). Structurally, the molecule consists of a substituted fluorene core bearing a p‐chlorobenzylidene moiety at C‐9, along with an *N*‐(dibutylamino)butanol‐derived side chain attached to the exocyclic aminoalkyl substituent. Due to its extended aminoalkyl and aliphatic substituents, LUME is highly flexible and can adopt numerous low‐energy rotamers. This extensive conformational space influences the accuracy of ECD predictions, which depend critically on the quality and sampling of the low‐energy conformers. As seen in Figure 6A, the predicted ECD spectrum of (R)‐LUME exhibits a strong positive band around 210 nm, which is consistent with the positive signal observed experimentally for E1. In addition, the calculated negative features between 240‐ and 300‐nm agree qualitatively with the dominant negative bands observed in E1. Figure 6B shows complementary behavior, where the predicted ECD profile of (S)‐LUME closely matches the experimental spectrum of E2.

### 3.5 Molecular Docking

Molecular docking studies were performed to gain further insights into the potential interaction mechanisms between the LUME enantiomers and the CSPs. These simulations were carried out considering LUME in its neutral state, which represents its expected protonation state under the experimental polar organic mode conditions where no additives were used. It is important to note the inherent limitations of the present study, such as the exclusion of solvent effects (which are critical in enantioseparations) and the reliance on simplified CSPs models. As highlighted by Peluso and Chankvetadze [61], molecular modeling in enantioseparation science still faces several challenges; consequently, the findings should be interpreted as supportive hypotheses rather than mechanisms proofs.

The docking studies performed using CCDC GOLD with the ChemPLP scoring function suggest that LUME may exhibit a higher affinity for Chirobiotic T and Chirobiotic V, with docking score values around 70 (Table 3). Although the score values are dimensionless and the docking does not account for solvent effects, in this case EtOH, the observed results may help us to hypothesize the inability of these two CSPs to separate the enantiomers. In contrast, LUME showed lower docking scores in Chiralpak ad, ranging from 51.73 to 57.43, suggesting weaker interactions with the CSP but still allowing enantiomeric separation.

**TABLE 3:** Molecular docking scores obtained for the best poses of LUME using ChemPLP scoring function.

Compound	Chiralpak ad	Chirobiotic T	Chirobiotic V
R‐LUME neutral	57.4263	72.9407	63.701
S‐LUME neutral	51.7337	70.7773	72.0432

**FIGURE 7:**
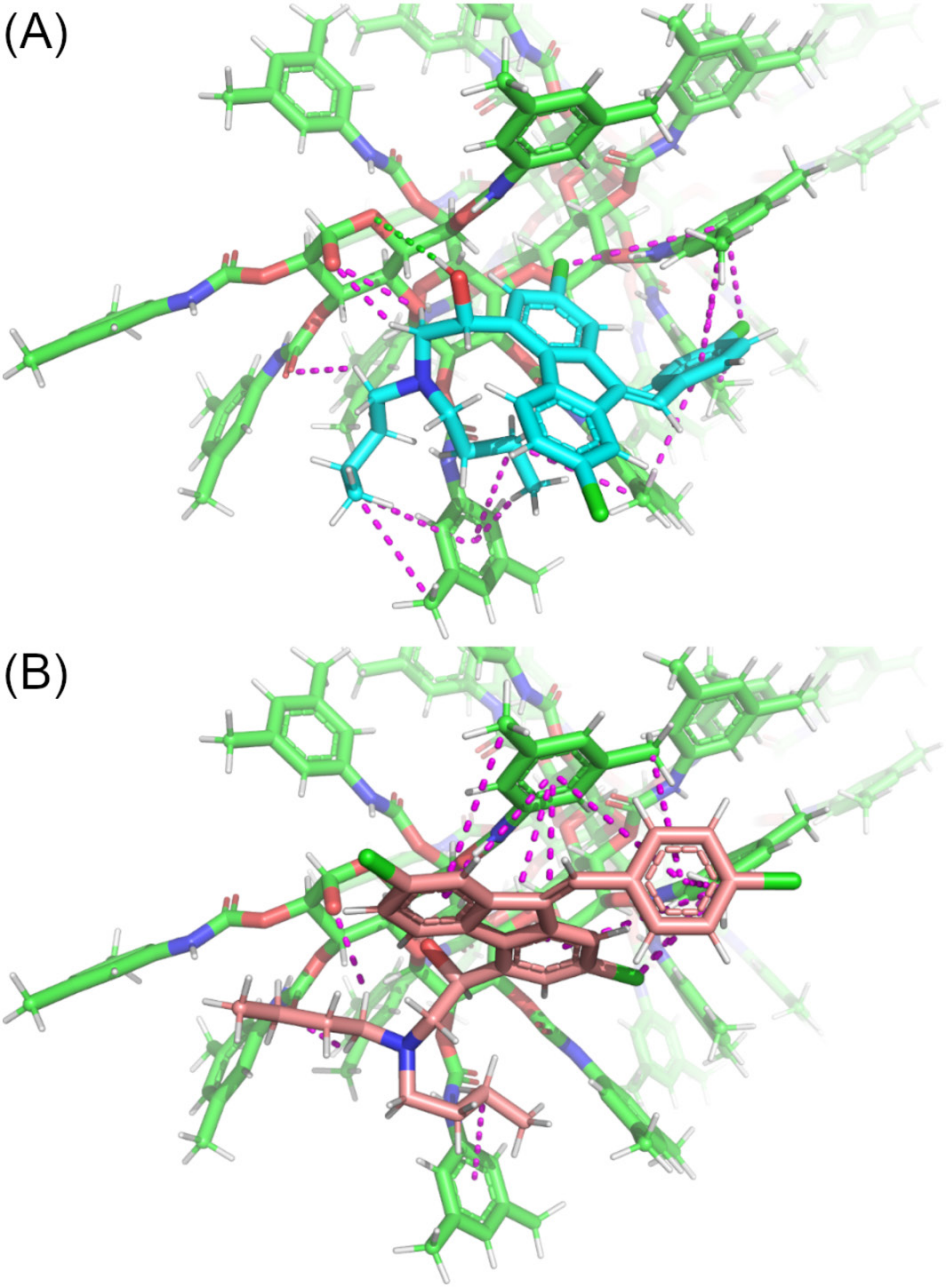
Interaction profile obtained for the best poses of LUME enantiomers in Chiralpak ad. (A) (R)‐LUME neutral state, (B) (S)‐LUME neutral state. The molecular interactions were represented in dashed lines with the following color scheme: magenta for van der Waals and π‐interactions and green for hydrogen bonds.

The analysis of the docking poses of LUME on Chiralpak ad showed a rotation and translation in the fluorene scaffold between R‐LUME and S‐LUME (Figure 7A,B, respectively). However, the compound remained docked within the same region and preserved most interactions with the CSPs. The best poses obtained on Chirobiotic T showed a similar pattern to Chiralpak ad, with a rotation of the fluorene core observed when comparing R‐LUME to S‐LUME (Figure S2). Finally, the analysis of LUME's best poses on Chirobiotic V revealed that, although the docking scores differed, the drug adopted similar poses. The fluorene core remained stable, and the main differences were observed in the benzylidene group and the lateral *N*‐(dibutylamino)butanol‐derived side chain (Figure S3).

Overall, the molecular interactions observed for LUME in all CSPs fall into three categories: (a) van der Waals and π‐interactions (represented as dashed magenta lines), mainly due to the bulky tricyclic ring (fluorene) and the p‐chlorobenzylidene group and (b) hydrogen bonds (dashed green lines) involving the hydroxyl group. Most interactions for LUME with Chiralpak ad were van der Waals and π‐interactions, whereas the other CSPs showed additional hydrogen bonds, increasing their interaction with LUME. These findings support the hypothesis that although LUME may mainly interact through hydrophobic interactions with all CSPs, the additional hydrogen bonding observed with the macrocyclic glycopeptide‐based CSPs may contribute to their failure to separate the enantiomers under the tested conditions. Furthermore, the enantioseparation is not solely determined by interaction strength but also depends on the geometric fit and spatial compatibility between LUME and the CSP [61].

## 4 Conclusion

There is a growing need to develop and adopt more environmentally friendly and sustainable approaches in pharmaceutical analysis. In this work, we developed a green chiral HPLC method for the determination of LUME enantiomers. Chiral separation was successfully achieved in 12 min using EtOH as the mobile phase. EtOH is considered a green solvent, can be obtained from a bio‐based source, is readily biodegradable, and has acceptable toxicity properties. This study demonstrates that hazardous solvents like hexane can be replaced with greener alternatives, such as EtOH, without compromising separation efficacy. The environmental sustainability of the proposed method was assessed with the AGREE and AGSA metrics, demonstrating favorable greenness attributes. The method was validated and can be applied to the quantification of LUME enantiomers in drug substance and pharmaceutical dosage form. Furthermore, the method can be extrapolated to preparative purposes to obtain the enantiopure forms. The ECD spectra calculated using the sTD‐DFT method proposed the R‐enantiomer as the first‐eluted peak. The molecular docking studies were consistent with the experimental observations, as the lowest docking scores were obtained for Chiralpak ad. To the best of our knowledge, this is the first report on ECD spectra prediction of LUME and molecular docking on these CSPs. This work contributes to the development of more sustainable chiral HPLC methods, supporting their application in enantioselective studies and pharmaceutical quality control.

## Supporting information


**Table S1:** Molecular docking binding site parameters according to the CSP structure.
**Figure S1:** UV spectrum of lumefantrine in ethanol.
**Figure S2:** Interaction profile obtained for the best poses of LUME enantiomers in Chirobiotic T. (A) (R)‐LUME neutral state; (B) (S)‐LUME neutral state. The molecular interactions were represented in dashed lines with the following color scheme: magenta for van der Waals and π‐interactions and green for hydrogen bonds.
**Figure S3:** Interaction profile obtained for the best poses of LUME enantiomers in Chirobiotic V. (A) (R)‐LUME neutral state; (B) (S)‐LUME neutral state. The molecular interactions were represented in dashed lines with the following color scheme: magenta for van der Waals and π‐interactions and green for hydrogen bonds.

## Data Availability

Data are available on request from the authors.
