# Supplementary material for: Green Chiral HPLC Method for Lumefantrine Analysis: Method Development, Greenness Assessment, and Computational Studies
Source: Chirality. 2026 Jul 16;38(8):e70122. doi: 10.1002/chir.70122 (PMC13373994; doi:10.1002/chir.70122)
Supplement: Supplementary file 1 — Table S1: Molecular docking binding site parameters according to the CSP structure. Figure S1: UV spectrum of lumefantrine in ethanol. Figure S2: Interaction profile obtained for the best poses of LUME enantiomers in Chirobiotic T. (A) (R)‐LUME neutral state; (B) (S)‐LUME neutral state. The molecular interactions were represented in dashed lines with the following color scheme: magenta for van der Waals and π‐interactions and green for hydrogen bonds. Figure S3: Interaction profile obtained for the best poses of LUME enantiomers in Chirobiotic V. (A) (R)‐LUME neutral state; (B) (S)‐LUME neutral state. The molecular interactions were represented in dashed lines with the following color scheme: magenta for van der Waals and π‐interactions and green for hydrogen bonds. [file CHIR-38-e70122-s001.docx]

**Supplementary Material**

Green chiral HPLC method for Lumefantrine analysis: method development, greenness assessment and computational studies

Amanda Mohr^a^*, Gustavo Machado das Neves*^b^*, Érika Segala*^c^*, Vera Lucia Eifler-Lima*^b^*, Martin Steppe^a^

*^a^Laboratório de Controle de Qualidade Farmacêutico (LCQFar), Faculdade de Farmácia, Universidade Federal do Rio Grande do Sul, Av. Ipiranga 2752, Porto Alegre, RS, Brasil*

*^b^Laboratório de Síntese Orgânica Medicinal (LaSOM), Faculdade de Farmácia, Universidade Federal do Rio Grande do Sul, Av. Ipiranga 2752, Porto Alegre, RS, Brasil*

*^c^Faculdade de Farmácia, Universidade Federal do Rio Grande do Sul, Av. Ipiranga 2752, Porto Alegre, RS, Brasil*

*Correspondence author e-mail: amandamohr21@hotmail.com

**Table S1 -**  Molecular docking binding site parameters according to the CSP structure.

| **CSP structure** | **Grid Location (XYZ)** | **CCDC GOLD**  **Grid radius (Å)** |
| --- | --- | --- |
| Chirobiotic T | -24.763388 -44.127322 -13.823237 | 10 |
| Chirobiotic V | 3.159258 8.632966 1.802899 | 10 |
| Chiralpak AD | 19.978451 20.073423 26.036014 | 30 |


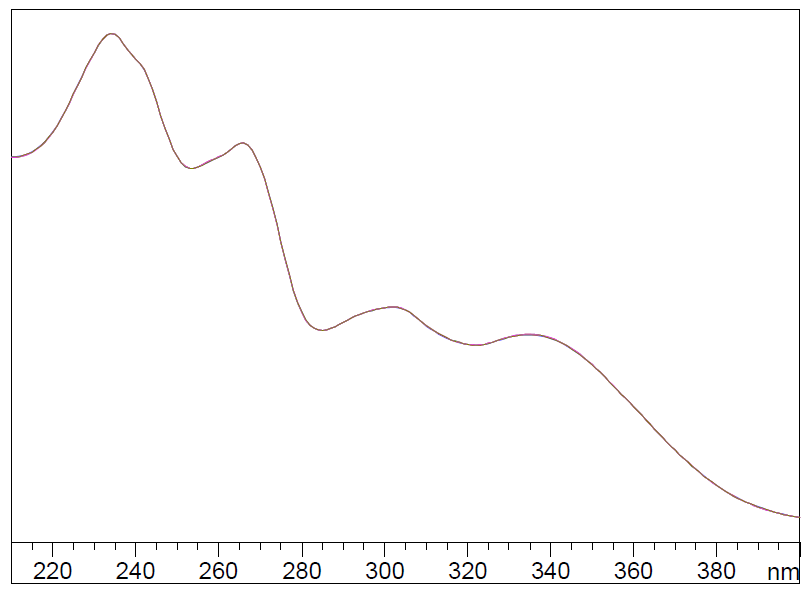


**Figure S1 -** UV spectrum of Lumefantrine in ethanol.


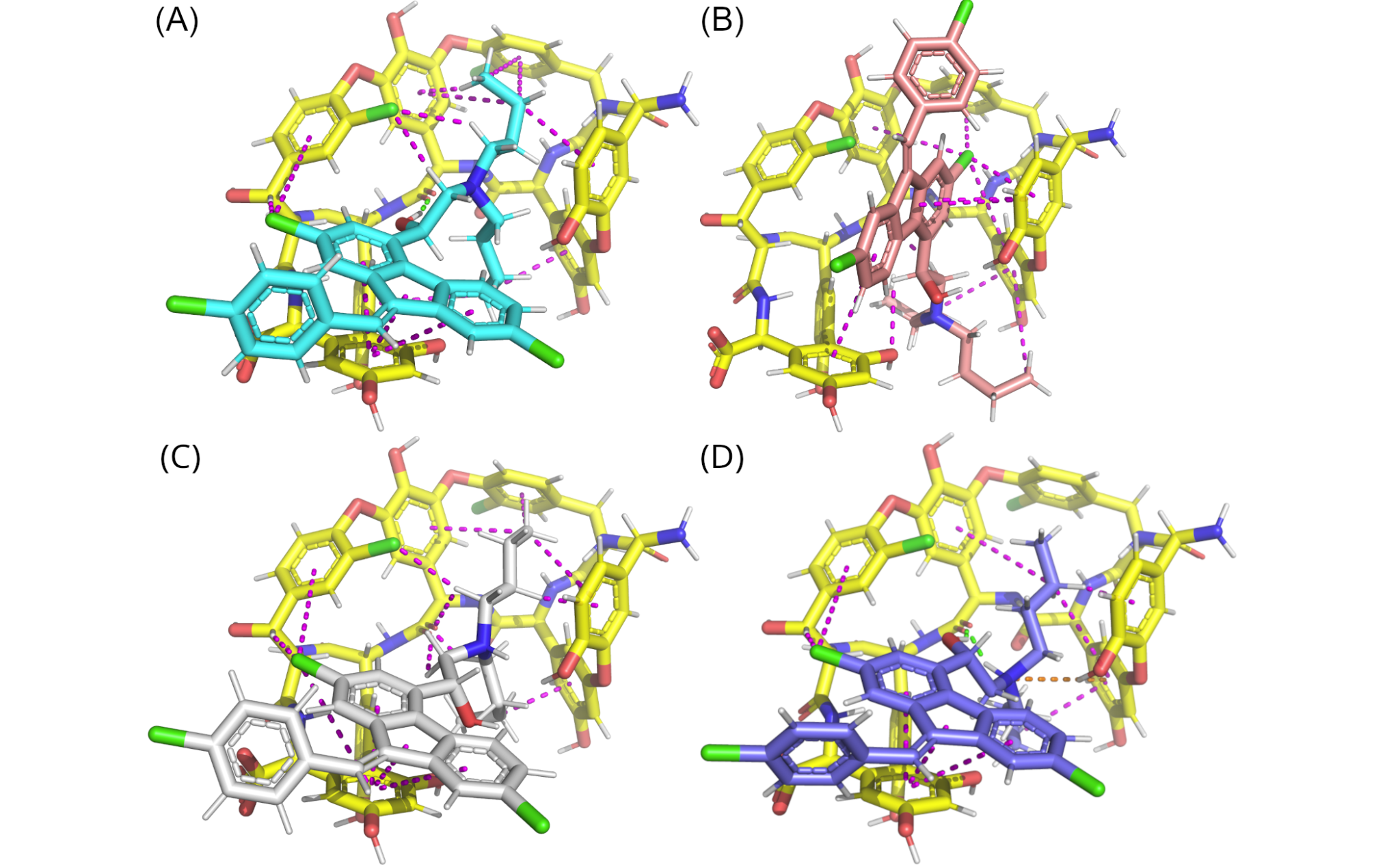


**Figure S2 -** Interaction profile obtained for the best poses of LUME enantiomers in Chirobiotic T. (A) (R)-LUME neutral state, (B) (S)-LUME neutral state. The molecular interactions were represented in dashed lines with the following colour scheme: magenta for Van der Waals and π-interactions and green for hydrogen bonds.


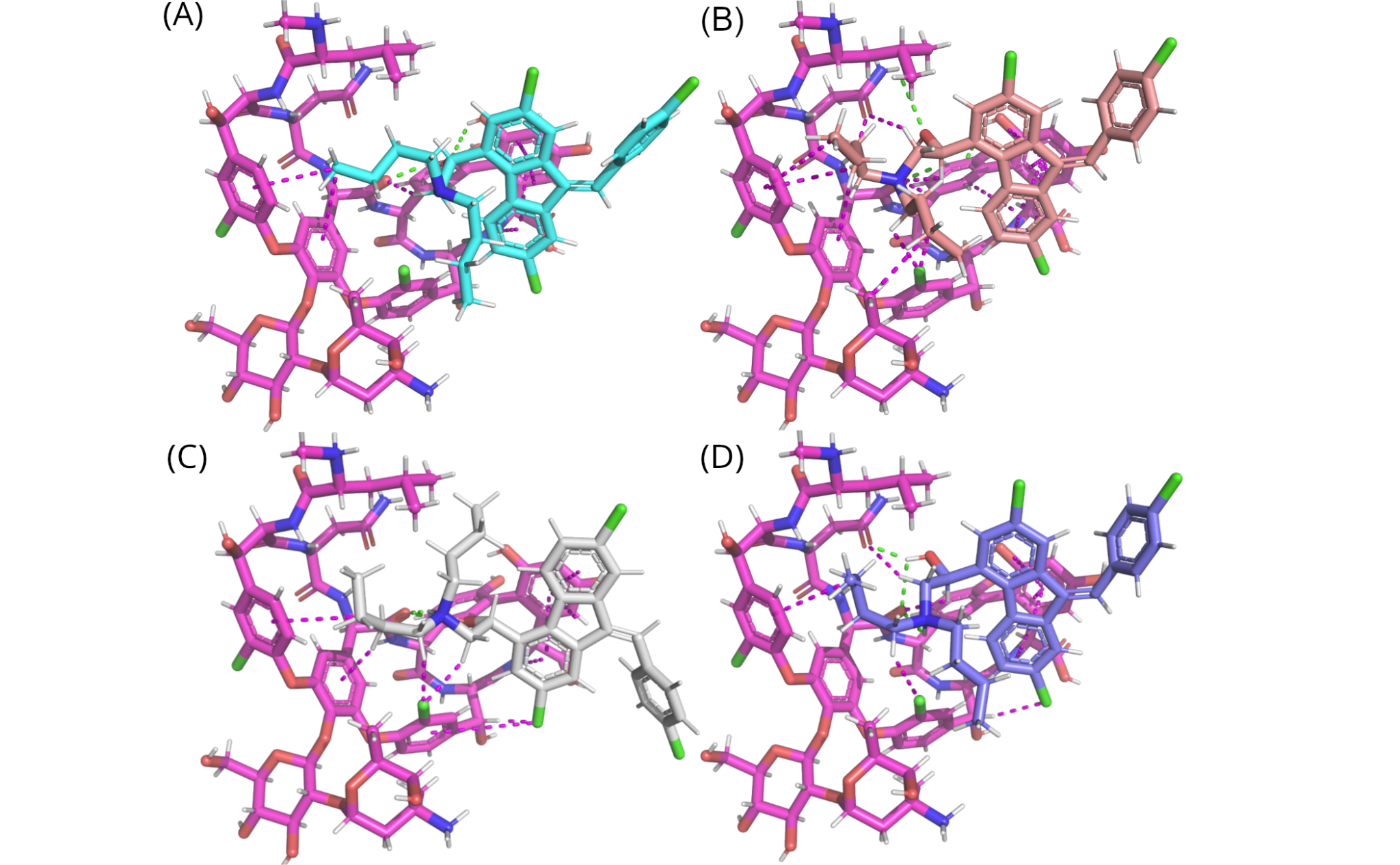


**Figure S3 -** Interaction profile obtained for the best poses of LUME enantiomers in Chirobiotic V. (A) (R)-LUME neutral state, (B) (S)-LUME neutral state. The molecular interactions were represented in dashed lines with the following colour scheme: magenta for Van der Waals and π-interactions and green for hydrogen bonds.
